# Novel intragenic deletions within the *UBE3A* gene in two unrelated patients with Angelman syndrome: case report and review of the literature

**DOI:** 10.1186/s12881-017-0500-x

**Published:** 2017-11-21

**Authors:** Cinthia Aguilera, Marina Viñas-Jornet, Neus Baena, Elisabeth Gabau, Concepción Fernández, Nuria Capdevila, Sanja Cirkovic, Adrijan Sarajlija, Marijana Miskovic, Danijela Radivojevic, Anna Ruiz, Miriam Guitart

**Affiliations:** 1 Genetics Laboratory, UDIAT-Centre Diagnòstic, Parc Taulí Hospital Universitari, Institut d’Investigació i Innovació Parc Taulí I3PT, Universitat Autònoma de Barcelona, Parc del Taulí 1, 08208 Barcelona, Sabadell Spain; 2 Paediatric Unit, Parc Taulí Hospital Universitari, Institut d’Investigació i Innovació Parc Taulí I3PT, Universitat Autònoma de Barcelona, Sabadell, Spain; 3 Laboratory for Medical Genetics, Mother and Child Health Care Institute of Serbia “Dr Vukan Cupic”, Belgrade, Serbia; 4 Department of Metabolism and Clinical Genetics, Mother and Child Health Care Institute of Serbia “Dr Vukan Cupic“, Belgrade, Serbia; 5 School of Medicine, University of Belgrade, Belgrade, Serbia

**Keywords:** Angelman syndrome (AS), *UBE3A*, Intragenic deletions, MLPA

## Abstract

**Background:**

Patients with Angelman syndrome (AS) are affected by severe intellectual disability with absence of speech, distinctive dysmorphic craniofacial features, ataxia and a characteristic behavioral phenotype. AS is caused by the lack of expression in neurons of the *UBE3A* gene, which is located in the 15q11.2-q13 imprinted region. Functional loss of *UBE3A* is due to 15q11.2-q13 deletion, mutations in the *UBE3A* gene, paternal uniparental disomy and genomic imprinting defects.

**Case presentation:**

We report here two patients with clinical features of AS referred to our hospital for clinical follow-up and genetic diagnosis. Methylation Specific-Multiplex Ligation-Dependent Probe Amplification (MS-MLPA) of the 15q11.2-q13 region was carried out in our laboratory as the first diagnostic tool detecting two novel *UBE3A* intragenic deletions. Subsequently, the MLPA P336-A2 kit was used to confirm and determine the size of the *UBE3A* deletion in the two patients. A review of the clinical features of previously reported patients with whole *UBE3A* gene or partial intragenic deletions is presented here together with these two new patients.

**Conclusion:**

Although rare, *UBE3A* intragenic deletions may represent a small fraction of AS patients without a genetic diagnosis. Testing for *UBE3A* intragenic exonic deletions should be performed in those AS patients with a normal methylation pattern and no mutations in the *UBE3A* gene.

## Background

Angelman syndrome (AS) is a neurogenetic disorder characterized by a severe intellectual disability with absence of speech, distinctive dysmorphic craniofacial features such as microcephaly with flat occiput and occipital groove, wide mouth, ataxia related neurological problems and/or tremor in the limbs and seizures with specific EEG pattern abnormalities that persist into adulthood. The behavioural phenotype is characterized by happy disposition, hyperactivity, attention deficit and frequent disruption of sleep cycles. Other clinical problems are gastrointestinal difficulties with feeding problems, gastroesophageal reflux, constipation, scoliosis, and an increased sensitivity to heat. Its prevalence is about 1/15000 births [1–3].

The cause of AS is the loss of function in neurons of the ubiquitin protein ligase E6-AP (E6-Associated Protein) encoded by the *UBE3A* gene, which is located on chromosome 15q11.2-q13 region. This region includes a cluster of genes under imprinting control which show differential expression depending on the parental origin, in a tissue-specific manner. *UBE3A* shows a specific expression of the maternal allele in neurons while the paternal allele is silenced by the synthesis of an antisense transcript from the *SNURF-SNPRN* gene (*UBE3A-ATS*). The absence of the specific maternal expression of *UBE3A* is the main cause of AS [4].

The functional loss of *UBE3A* in the maternal allele is due to different genetic mechanisms: (i) deletion of the region 15q11.2-q13 in 70–75% of patients, (ii) mutations in the *UBE3A* gene in 10–15% of the patients, (iii) paternal uniparental disomy in 1–3% of patients, and (iv) genomic imprinting defects in 2–4% of the patients. The genetic cause remains unknown in approximately 10% of patients presenting clinical features characteristic of AS [4, 5]. There are also some reports that describe a proportion of AS patients who harbor microdeletions of the *UBE3A* gene [6–10].

The *UBE3A* gene spans 120 kb of genomic DNA and consists of 10 exons (NM_130838.1). The ubiquitin protein ligase E6-AP encoded by the *UBE3A* gene is necessary for the ubiquitination of proteins targeted for degradation. E6-AP belongs to the HECT (homologous to E6-AP COOH terminus) class of E3 enzymes that share a 40 kDa COOH-terminal catalytic domain. This domain is encoded by exons 3–10 [11]. Exons 1 to 5 encode a steroid co-activation region which has several LXXLL motifs that are known to be receptor interacting motifs [12].

Mutations in the *UBE3A* gene are widely distributed and have been detected throughout all regions of the gene [13, 14]. Rare intragenic deletions and duplications may represent a higher percentage of mutations than expected, because these alterations escape the routine screening [15].

Here, we report two patients with AS who present two novel intragenic deletions within the *UBE3A* gene.

## Case presentation

*Patient 1* is a 5 year-old girl born at 37 weeks of gestation. Birth weight was 2850 g. Neither perinatal problems nor hypotonia was reported. Initially, she didn’t show feeding problems but when solid food was introduced into her diet, she had difficulties to swallow. Psychomotor development was delayed. She sat unsupported at 9 months, walked independently at 24 months and pronounced only two words which she lost later. She came to the clinical geneticist when she was 2-year-8-month old. Phenotypic features included relative microcephaly (47 cm, −1.75 SD), small and wide-spaced teeth and protruding tongue. Her weight was 12 Kg (25th percentile) and her height was 80 cm (50th percentile). Neurological examination detected severe intellectual disability, ataxia of gait, receptive and non-verbal communication skills higher than verbal ones, hyperreflexia of the lower extremities, tremulous movement of limbs and frequent drooling. She also had abnormal sleep-wake cycles and by the age of 20 months she developed seizures. EEG showed generalized slow wave activity with paroxysmal activity. The behavioural phenotype included frequent laugher, happy demeanour, easily excitable personality, hyperactive behaviour, attention deficit and exploration of objects throughout the mouth. She also presented attraction to water and an increased sensitivity to heat (Table 1).

**Table 1:** Clinical data of patients with *UBE3A* intragenic deletions, following the clinical features of AS described by Williams et al., 2006

	Patient 1	Patient 2	Bürger, et al., 2002	Boyes, et al., 2006	Boyes, et al., 2006	Calì, et al., 2010	Beleza-Meireles, et al., 2011	Piard, et al., 2011	Piard, et al., 2011
Genetics									
Intragenic deletion	Exon 2^a^	Exons 9-10^a^	Whole *UBE3A*	Exons 8-16^b^	Exons 8-16^b^	Exon 8^c^	Exons 5-12^c^	Exons 6-12^c^	Exons 6-12^c^
Predicted protein	p.0?	p.Arg765_852del88	p. 0?	p.0?	p.0?	p.Leu517Valfs^a^27	p.0?	p.0?	p.0?
Inheritance	De novo	De novo	Maternal	Maternal	Maternal	Maternal	NA	Maternal	Maternal
Clinical data									
Sex	F	F	M	F	M	M	F	F	F
Weight at birth	2850 g	3000 g	2670 g	3200 g	3350 g	3370 g	3880 g	NA	NA
Perinatal problems	–	–	NA	NA	NA	–	–	NA	NA
Age of sedestation (months)	9	8	12	18	21	18	NA	9	NA
Age of walk (months)	24	24	18	39	39	24	24	24	24
Consistent clinical features of AS (100% of the affected individuals)									
Ataxia of gait	+	+	+	+	+	+	+	NA	+
Frequent laugher/smiling	+	+	+	+	+	+	+	+	+
Apparent happy demeanor	+	+	NA	+	+	NA	+	+	+
Easily excitable personality	+	+	NA	NA	NA	NA	NA	NA	NA
Hyperactive behaviour	+	+	NA	+	+	NA	+	NA	+
Attention deficit	+	+	NA	NA	NA	NA	NA	NA	NA
Hand flapping/stereotipies	–	+	NA	+	NA	NA	+	+	+
Development delay	+	+	+	+	+	+	+	+	+
Severe mental retardation	+	+	+	+	+	+	+	+	+
Speech impairement	+	+	+	+	+	+	+	+	+
Receptive and non-verbal communication skills higher than verbal ones	+	+	+	NA	NA	NA	NA	NA	NA
Frequent clinical features of AS (more than 80% of the affected individuals)									
Microcephaly	Relative	–	+	+	+	+	–	+	+
Seizures	+	+	NA	+	+	–	+	+	+
Abnormal EEG	+	+	–	+	+	+	+	+	NA
Associatedclinical features of AS (20–80% of the affected individuals)									
Hypotonia	–	+	–	+	+	+	+	NA	NA
Feeding problems	–	–	–	NA	NA	NA	+	NA	NA
Prognathia	–	+	NA	NA	NA	NA	+	NA	NA
Flat Occiput	–	–	NA	NA	NA	NA	+	NA	NA
Occipital groove	–	–	NA	NA	NA	NA	NA	NA	NA
Hypopigmented skin, light hair and eye color	–	–	–	+	+	NA	NA	NA	NA
Strabismus	–	–	NA	NA	NA	NA	NA	+	+
Wide mouth	–	+	–	+	NA	NA	NA	+	NA
Wide-spaced teeth	+	+	–	NA	NA	NA	NA	+	+
Protruding tongue	+	–	–	+	NA	NA	NA	NA	NA
Small hands and feet	–	–	NA	NA	NA	NA	NA	NA	NA
Scoliosis	–	–	NA	NA	+	+	NA	NA	+
Uplifted flexed arm position, especially during deambulation	–	+	NA	NA	NA	NA	NA	NA	NA
Hyperreflexia of the lower extremities	+	–	NA	+	NA	+	NA	NA	NA
Tremolous movement of limbs	+	–	NA	NA	NA	NA	NA	NA	NA
Freqüent drooling	+	+	NA	NA	NA	+	NA	NA	NA
Suck/swallowing disorders	+	–	NA	NA	NA	NA	NA	NA	+
Abnormal sleep-wake cycle	+	–	NA	+	+	NA	NA	+	+
Chewing/mouthing behaviour	+	+	NA	NA	NA	NA	NA	NA	NA
Atraction to/fascination with water	+	–	NA	NA	NA	NA	NA	NA	NA
Increased sensitivity to heat	+	–	NA	NA	NA	NA	NA	NA	NA

*F* female, *M* male, *+* present, *−* absent, *NA* not available

^a^Nomenclature exons 1–10 according to NM_130838.1

^b^Nomenclature exons according to Kishino and Wagstaff, 1998; Yamamoto, et al., 1997

^c^Nomenclature exons 1–14 according to NM_00462.3

Methylation PCR and *UBE3A* sequencing were carried out in another centre with negative results. MS-MLPA analysis was performed in our laboratory using a specific kit, SALSA MS-MLPA ME028-B2 Prader Willi/Angelman, (MRC Holland, Amsterdam, The Netherlands). This kit contains 46 probes, 32 of which are specific for sequences in or close to the PWS/AS critical region on 15q11.2-q13 which can be used to detect copy number changes in this region. As a control for copy number changes, 14 probes outside the PWS/AS region are included. Among the PWS/AS specific probes, seven probes are methylation-sensitive and contain a *Hha*I restriction site. Five probes are located within the *UBE3A* gene corresponding to exons 1, 2, 3, 4 and 9 (NM_130838.1). In this paper, we use exon numbering according to NM_130838.1 sequence which is the standard reference sequence for the *UBE3A* gene where exons 1 to 10 correspond to exons 5b to 14 of the NM_000462.3 sequence. MLPA was performed according to manufacturer’s instructions (MRC Holland). Amplification products were run on an ABI3130 analyser (Applied Biosystems, California, USA) and analyzed using the GeneMapper software (Applied Biosystems).

**Fig. 1:**
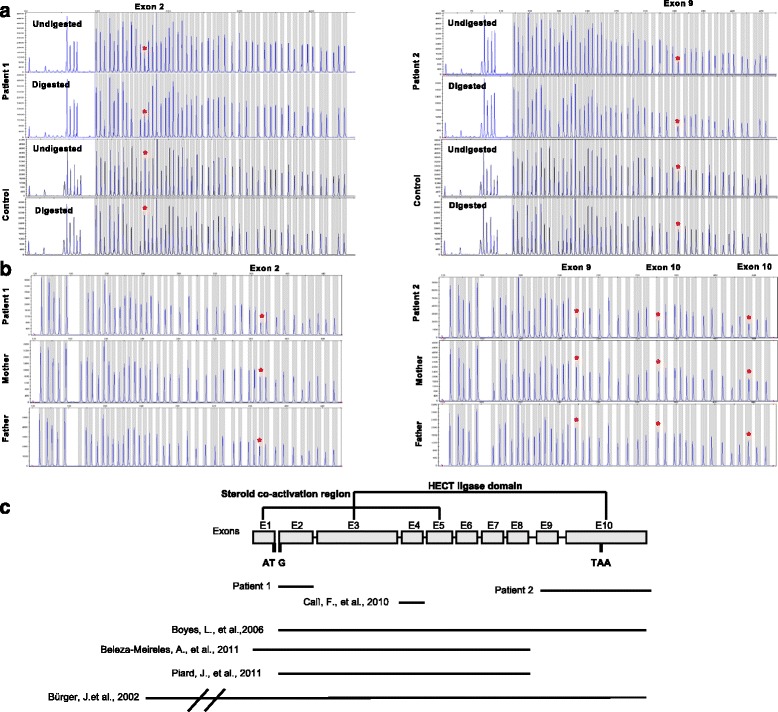
Two intragenic deletions within the *UBE3A* gene (NM_130838.1) were identified by MLPA analysis in patients 1 and 2. **a** MS-MLPA analysis with the ME028-B2 kit shows a normal methylation pattern and a heterozygous deletion of exon 2 in patient 1. A normal methylation pattern and a heterozygous deletion of exon 9 were observed in patient 2. Red asterisks indicate the deleted exons. **b** MLPA analysis with the kit P336-A2 confirmed the deletion of exon 2 in patient 1 while a deletion involving exon 9 and 10 was observed in patient 2. In both cases the deletions were de novo. Red asterisks indicate the deleted exons. **c** Summary of *UBE3A* intragenic deletions identified in this study and the literature. Exons are depicted as grey boxes. Exons 1 to 5 constitute the steriod co-activation region and exons 3 to 10 constitute the HECT ligase domain, according to Ramamoorthy and Nawaz et al., 2008. *UBE3A* deletions reported here and in the literature are shown as black bars

Methylation pattern of the 15q11.2-q13 region was normal. However, dosage analysis showed a reduction of 50% in the relative peak height of the probe corresponding to *UBE3A* exon 2 while probes corresponding to *UBE3A* exons 1, 3, 4 and 9 (NM_130838.1) showed a normal diploid dosage (Fig. 1a). To confirm the specific *UBE3A* exon 2 deletion the MLPA P336-A2 kit (MRC Holland) was used. This kit contains 42 MLPA probes, including at least one probe for each exon of the *UBE3A* gene*.*

A reduction of 50% in the relative peak height corresponding to the probe located in the exon 2 was detected while all other *UBE3A* exonic probes presented normal dosage confirming that the deletion only involved exon 2 (Fig. 1b). Parents testing showed that deletion was de novo (Fig. 1b), although a germline mosaic cannot be excluded. In order to investigate in which parental allele the deletion was originated, we used six SNP located in intron 2: rs71418040, rs17114442, rs28754450, rs57111857, rs757438 and rs61336305. All of them were not informative so the maternal origin of the deletion could not be confirmed.

*Patient 2* is a 5 year-old girl born at term following an uneventful pregnancy. Her birth weight was 3000 g. No perinatal problems were reported but she was noted to be hypotonic. She presented developmental delay. Unsupported sedestation was achieved at 8 months and independent walking at 24 months. Oral language was limited to around 10 words.

Physical features of the patient included prognathia, wide mouth and wide-spaced teeth. The neurological manifestations included severe intellectual disability, ataxia of gait, uplifted and flexed arm position during deambulation, speech impairment, frequent drooling and seizures. The EEG showed generalized slow wave activity with paroxysmal activity. She didn’t present disturbances of the sleep-wake cycle.

The behavioral traits included a happy demeanor with smiling, laughing, hyperactive behavior, attention deficit, hand flapping and chewing/mouthing behaviors. At the age of the diagnosis, 5.6-year-old, her weight was 15 Kg (10th percentile) and the head circumference was 48 cm (−1.5 SD) (Table 1).

Initially, MLPA analysis was carried out using the SALSA MLPA P064 Mental Retardation probemix-1 in the Mother and Child Health Care Institute of Serbia. The results showed a deletion of the exon 10 (NM_130838.1) of the *UBE3A* gene (data not shown). Subsequently, a DNA sample of the patient was sent to our laboratory for further studies. MS-MLPA testing was performed and showed a normal methylation pattern. However, a reduction of 50% in the relative peak height of the probe corresponding to the *UBE3A* exon 9 was observed while probes corresponding to *UBE3A* exons 1, 2, 3, 4 (NM_130838.1) showed a normal diploid dosage (Fig. 1a). To confirm the *UBE3A* exon 9 and 10 deletion and to determine whether the deletion expanded to other exons, MLPA P336-A2 kit was used. MLPA analysis confirmed the deletion only involving exon 9 and 10 (Fig. 1b). The genetic analysis in the parents showed that the deletion encompassing exons 9 and 10 was de novo (Fig.1b). In patient 2, two SNPs in intron 9: rs573271880, rs17115485 and 1 SNP in exon 10: rs10047992 were analyzed to investigate the parental origin of the deletion. As in patient 1, we could not confirm the maternal origin of the deletion.

## Discussion and conclusions

Angelman syndrome is caused by the lack of expression of the maternal copy in neurons of the *UBE3A* gene due to four different molecular etiologies: Deletion of the 15q11.2-q13 region on the maternal chromosome, mutations in the maternally-inherited copy of *UBE3A,* paternal uniparental disomy for chromosome 15 and an imprinting defect causing lack of expression of the maternal copy of *UBE3A* [16].

Genotype-phenotype correlations among molecular subclasses have shown that deletion patients show a more severe phenotype in all aspects of neurodevelopmental delay except for expressive language skills that are extremely poor regardless of their molecular subclass [17–21]. However, Mertz et al., 2014 report that children with AS due to an *UBE3A* mutation or paternal uniparental disomy present significantly better linguistic properties than deletion patients, in particular the expressive area, where 6 out of 9 children with *UBE3A* mutation or paternal uniparental disomy could use 2–7 words while it was just the case in 3 out of 30 children with a 15q11.2-q13 deletion [22]. In addition, the deletion class is the most severely affected regarding microcephaly, seizures, relative hypopigmentation, motor difficulties while paternal uniparental disomy and imprinting defect individuals have better physical growth, have less movement and ataxia abnormalities, and have a lower prevalence (but not absence) of seizures [18]. In particular, individuals with deletions manifest higher rates of epilepsy (90%, compared to 75% among individuals without deletion), earlier onset of seizures (mean of age 1.9 years, compared with 6.3 years among individuals without deletion) and may exhibit a more severe electroencephalogram phenotype compared to the other etiologies [23].

It has been suggested that AS patients carrying *UBE3A* mutations show a phenotype somewhere in the middle between deletion and paternal uniparental disomy carriers. They present higher incidences of seizures and microcephaly, similar to deletion patients, while their neurodevelopment delay is similar to paternal uniparental disomy and imprinting defect patient carriers [18, 19]. A revision of genotype-phenotype differences has been published recently by La Salle et al. [24].

The patients presented in this report carry intragenic *UBE3A* exonic deletions that affect the *UBE3A* open reading frame. Exon 2 deletion in patient 1 is predicted to eliminate the start codon and consequently no protein will be produced (p.0?). Deletion of exons 9 and 10 in patient 2 is predicted to eliminate the last 88 aminoacids (p.Arg765_Leu852del88) disrupting the ubiquitin ligase catalytic domain located at the 3′ end of the protein (Fig. 1c).

So far, seven patients with whole *UBE3A* gene or partial intragenic deletions have been reported (Fig. 1c). A summary of their clinical features is presented in Table 1. All deletions reported previously are also predicted to affect the *UBE3A* open reading frame and create a premature stop codon at the beginning of the protein (Table 1). Only patient 2 and the patient reported by Cali et al., 2010 present exon deletions that would alter the 3′ end of the *UBE3A* disrupting the ubiquitin ligase activity of the protein.

All patients show the consistent clinical features of AS except for patient 1 who does not present stereotypes. This is surprising because stereotypes have been described as a clinical feature often present in all patients with AS since early in development. Microcephaly is present in almost all patients (7 of nine patients) while seizures are present in all except one (7 of eight patients available). The high incidence of seizures and microcephaly is in accordance to what has been reported before by Lossie et al., 2001 and Moncla et al., 1999. Associated clinical features show variability between patients. A comparison of AS patients with mutations and intragenic deletions has not been reported before and from our observations there are no differences between the two groups.

In our laboratory, array Comparative Genomic Hybridization (aCGH) is the first diagnostic tool in neurodevelopmental disorders. Methylation Specific-Multiplex Ligation-Dependent Probe Amplification (MS-MLPA) of the 15q11.2-q13 region is carried out when there is a strong clinical suspicion of Angelman or Prader Willi syndrome. MS-MLPA analysis simultaneously assesses the methylation status and genomic dosage changes at the 15q11.2-q13 region and can confirm the diagnosis and identify the presence of a causative deletion in 70% of cases. In the case of a normal methylation pattern and if the clinical suspicion remains high, sequence analysis of the *UBE3A* gene is performed, as 10% of Angelman patients harbor *UBE3A* mutations.

In addition, we propose here that the analysis of the copy number variations within the *UBE3A* gene should be taken into account, once the MS-MLPA test and the sequencing of *UBE3A* have been carried out with a negative result. Although rare, intragenic deletions may account for a small proportion of AS patients without a genetic diagnosis and if present, they have a recurrence risk of up to 50%, depending on the carrier status of the mother. In the two patients reported here the mutation has appeared de novo but in the other intragenic deletions previously reported they were transmitted by healthy mothers to their affected children (Table 1). Moreover, MLPA analysis of the *UBE3A* gene can be used to confirm and precisely establish the size of an intragenic deletion detected by MS-MLPA. The SALSA MLPA kit P336-A2 *UBE3A* contains probes for all coding exons of the *UBE3A* gene (exons 1–10, NM_130838.1).

To date, few reports have evaluated the contribution of exonic deletions to the spectrum of *UBE3A* mutations. Here, we report two patients with AS who present two novel intragenic deletions within the *UBE3A* gene that together with those reported in the literature show that *UBE3A* intragenic deletions may represent a small fraction of AS patients. Testing for *UBE3A* intragenic exonic deletions/duplications should be included in those AS patients with a normal methylation pattern and no mutations in the *UBE3A* gene.

## Abbreviations

AS: Angelman syndrome
E6-AP: E6- Associated Protein
HECT: Homologous to E6-AP COOH terminus
MLPA: Multiplex Ligation-Dependent Probe Amplification
MS-MLPA: Methylation Specific Multiplex Ligation-Dependent Probe Amplification
